# Entropic stabilization of a deubiquitinase provides conformational plasticity and slow unfolding kinetics beneficial for functioning on the proteasome

**DOI:** 10.1038/srep45174

**Published:** 2017-03-24

**Authors:** Yun-Tzai Cloud Lee, Chia-Yun Chang, Szu-Yu Chen, Yun-Ru Pan, Meng-Ru Ho, Shang-Te Danny Hsu

**Affiliations:** 1 Institute of Biological Chemistry, Academia Sinica, Taipei, 11529, Taiwan; 2 Institute of Biochemical Sciences, National Taiwan University, 10617, Taiwan

## Abstract

Human ubiquitin C-terminal hydrolyase UCH-L5 is a topologically knotted deubiquitinase that is activated upon binding to the proteasome subunit Rpn13. The length of its intrinsically disordered cross-over loop is essential for substrate recognition. Here, we showed that the catalytic domain of UCH-L5 exhibits higher equilibrium folding stability with an unfolding rate on the scale of 10^−8^ s^−1^, over four orders of magnitudes slower than its paralogs, namely UCH-L1 and -L3, which have shorter cross-over loops. NMR relaxation dynamics analysis confirmed the intrinsic disorder of the cross-over loop. Hydrogen deuterium exchange analysis further revealed a positive correlation between the length of the cross-over loop and the degree of local fluctuations, despite UCH-L5 being thermodynamically and kinetically more stable than the shorter UCHs. Considering the role of UCH-L5 in removing K48-linked ubiquitin to prevent proteasomal degradation of ubiquitinated substrates, our findings offered mechanistic insights into the evolution of UCH-L5. Compared to its paralogs, it is entropically stabilized to withstand mechanical unfolding by the proteasome while maintaining structural plasticity. It can therefore accommodate a broad range of substrate geometries at the cost of unfavourable entropic loss.

Ubiquitin C-terminal hydrolases (UCHs) are papain-like cysteine proteases that deubiquitinate by hydrolysing the isopeptide bond between the C-terminus of ubiquitin and the lysine side-chain of its adduct, countering ubiquitination in protein homeostasis^1^,^2^. Part of the much larger superfamily of deubiqutinases (DUBs), there are four UCHs in the human genome, namely UCH-L1, -L3, -L5 (also known as UCH37) and BAP1 (BRCA1-associated protein 1)^3^. While UCH-L1 and -L3 are single-domain proteins, UCH-L5 and BAP1 have an additional UCH37-like domain, ULD, at their C-termini. The DUB activity of UCH37 can be activated upon binding to a subunit of 26S proteasome, Rpn13^4^,^5^,^6^,^7^. As a proteasome-associated DUB, UCH-L5 acts at the last checkpoint of the ubiquitin-proteasome pathway (UPS) before the ubiquitinated substrates are degraded by the proteasome. Deubiquitination by UCH-L5 prevents ubiquitinated substrates from being degraded by the UPS and thus, this protein editing process can be likened to a tug of war between DUBs and UPS for maintaining protein homeostasis. Despite its importance, however, little is known about how and when UCH-L5 rescues specific substrates by exerting DUB activity.

While the UCH domain of UCH-L5 shares limited sequence homology with UCH-L1 (23%) and UCH-L3 (28%) compared to the much high sequence homology between UCH-L1 and -L3 (55%), their three-dimensional structures are essentially identical. The few differences in local dynamics result in some disordered secondary structures (Supplementary Results)^1^,^8^,^9^,^10^,^11^,^12^,^13^,^14^. In addition to the conserved catalytic triad, C90, H169, and D184 in UCH-L5, the residues lining the ubiquitin binding interface are also highly conserved, forming a deep crater to accommodate the ubiquitin C-terminus. Part of the ubiquitin-binding interface of UCHs is formed by the so-called cross-over loop^12^,^13^. The cross-over loop of UCH-L5 is six and three residues longer than that of UCH-L1 and -L3, respectively. The electron density of the cross-over loops and preceding α-helix 6 of UCH-L3 and UCH-L5 in their apo forms could not be resolved due to intrinsic disorder. The flexibility, however, is important for substrate recognition, as UCH-L5 is the only UCH amongst the three that is capable of hydrolysing K48-linked di-ubiquitin. Grafting the cross-over loop of UCH-L5 onto UCH-L1 renders the chimera capable of hydrolysing K48-linked di-ubiquitin, indicating that conformational plasticity of the ubiquitin binding pocket is critical for substrate recognition of UCHs^15^. Indeed, comparing the reported crystal structures of UCH-L5 in its apo forms and its co-factor bound forms reveals that the UCH-L5 catalytic domain folds into a well-defined structure with limited variation in terms of its overall structures under different states (Supplementary Results), though there exists a high degree of structural plasticity in the cross-over loop region as well as in α-helix 7 where ubiquitin makes direct contacts (Supplementary Results). In contrast, the impacts of ubiquitin binding to UCH-L1 and -L3 are mostly limited to the conformational rearrangements in the cross-over loops and flanking regions, and, in the case of UCH-L1, a cascade of side-chain movements to align catalytic residues into a productive configuration^9^,^13^.

Enzyme kinetics analyses of UCH-L1, -L3, and -L5 using a fluorogenic ubiquitin derivative, ubiquitin C-terminal 7-amido-4- methylcoumarin (UbAMC), as a model substrate indicate that UCH-L1 and -L3 form a productive Michelis-Menten complex with ubiquitin (*K*_M_ value) at a low nM range^16^,^17^,^18^,^19^, while that of UCH-L5 (a truncated form of residues 1–240 that retains the catalytic domain, hereafter denoted as UCH-L5_N240_)^18^ is three orders of magnitude higher, which may be attributed to the unfavourable entropic loss upon ubiquitin binding. Nonetheless, UCH-L5 exhibits a robust turnover rate (*k*_cat_) for which UbAMC that is over three orders of magnitude higher than that of UCH-L1 and slightly higher than that of UCH-L3^1^. Considering that UCH-L5 is associated with the proteasome, where the local concentration of ubiquitin is much higher than the average cellular concentration, UCH-L5 might effectively deubiquitinate its substrates for editing purposes before the substrates are degraded by the proteasome.

In addition to their potential role in protein homeostasis, UCHs contain a complex Gordian knot with five projected crossings in their folding topologies, rendering them an intriguing system for protein folding studies^20^,^21^. Knotted proteins can be found in all kingdoms of life and many knotted proteins are enzymes. The question of how knotted proteins attain their intricate knotted topologies have attracted the attention of experimental and computational biophysicists in recent years^22^. Remarkably, two bacterial RNA methyltransferases, YibK and YbeA, have been shown to knot spontaneously immediately after protein synthesis during de novo folding without the aid of chaperonins^23^, and they remain knotted in the presence of highly concentrated chemical denaturant^24^ while behaving like random coils^25^. The knotted structural element of bacterial RNA methyltransferases has been shown to be of functional importance in substrate recognition^26^. Indeed, the cross-over loop of UCHs is also essential in ubiquitin recognition despite the variations in loop length and sequence composition.

The folding dynamics and kinetics of UCH-L1 and -L3 were recently delineated^27^,^28^. Both UCHs exhibit two well-defined kinetic folding intermediates connected by two parallel folding pathways between the native and denatured states. We further demonstrated that UCH-L1 exhibits partially unfolded forms (PUFs) under native conditions that share structural characteristics with those of the chemically-induced intermediate states^28^. As part of our endeavour to systematically characterize the folding of knotted proteins^28^,^29^,^30^,^31^,^32^, particularly those within the same family, we applied the same experimental strategy for studying UCH-L1 and -L3 to investigate in detail the folding dynamics and kinetics of the UCH domain of UCH-L5. Our folding analyses indicate that UCH-L5 is thermodynamically more stable than UCH-L1 and -L3, with unfolding kinetics over four orders of magnitude slower than those of UCH-L1 and -L3. Despite the slow global unfolding kinetics, however, UCH-L5 displays abundant local fluctuations within its secondary structure elements manifested in rapid hydrogen-deuterium exchange (HDX). Our results therefore represent a rare example of an entropically stabilized biological system that requires a significant degree of structural plasticity for substrate recognition and accommodation in a hostile environment that is constantly subject to mechanical unfolding by the proteasome for protein degradation.

## Results

### Folding equilibrium of UCH-L5_N240_ monitored by intrinsic fluorescence and far-UV CD spectroscopy

**Figure 1:**
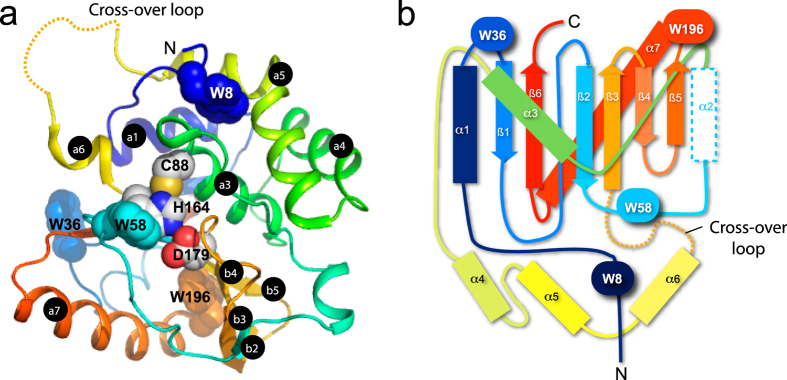
Three-dimensional structure of UCH-L5_N240_ and its knotted folding topology. (**A**) Cartoon representation of the crystal structure of UCH-L5_N240_ in complex with ubiquitin (PDB ID: 4UEL). UCH-L5_N240_ is colour-ramped from blue to red from the N- to C-termini and ubiquitin is coloured grey with its C-terminus shown in grey sticks. The side-chain atoms of the catalytic residues are shown in spheres with carbon, nitrogen, oxygen and sulphur atoms shown in white, blue, red and yellow, respectively, and their identities indicated accordingly. The tryptophan side-chains are shown in semi-transparent spheres. The cross-over loop was ill-defined due to its flexibility and is indicated in dashed gold line. (**B**) Topological representation of UCH-L5_N240_ illustrating the distribution of the tryptophan residues. The colouring scheme is the same as in (**A**). α-helices and β-strands are shown in rectangular and arrows, respectively.

**Figure 2:**
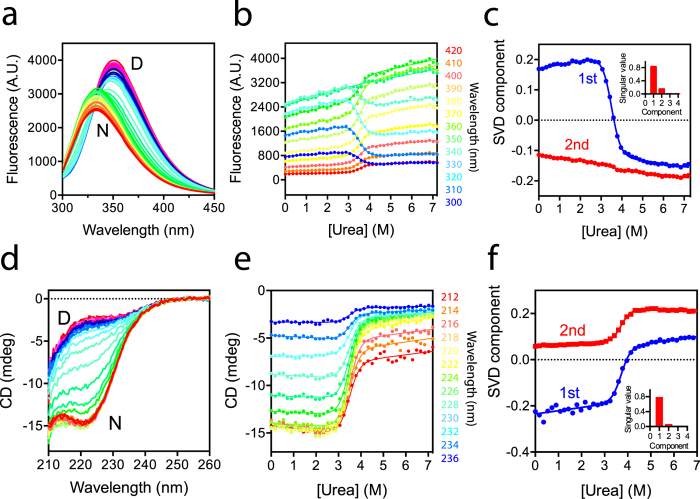
Urea-induced equilibrium unfolding of UCH-L5_N240_. (**A**) Intrinsic fluorescence spectra of UCH-L5_N240_ as a function of urea concentration, which are colour-ramped from red to purple from 0 to 7.2 M urea. (**B**) Global-fit of the urea-induced fluorescence change as a function of urea concentration at multiple wavelengths as indicated. (**C**) SVD analysis of the equilibrium unfolding data. The values of the first two SVD components are shown as a function of urea concentration and are coloured blue and red, respectively. Inset: The normalized contributions of the first four SVD components of which the first two account for more than 95% of the total signals. (**D**) Far-UV CD spectra of UCH-L5_N240_ as a function of urea concentration, which are presented in the same scheme as in (**A**). (**E**,**F**) Global-fit and SVD analyses of the far-UV CD data, presented in the same scheme as (**B**,**C**), respectively.

To compare the folding stability of UCH-L5_N240_ with that of UCH-L1^33^ and -L3^27^, we analysed its chemical stability by urea-induced equilibrium unfolding analysis through intrinsic fluorescence and far-UV CD spectroscopy. UCH-L5_N240_ has four tryptophan residues that are strategically distributed in the backbone topology that serve as ideal structural probes (Fig. 1). W8 and W36 are in proximity to the N- and C-termini that are involved in knot formation. W58 is adjacent to the catalytic site, encompassing C88, H164, and D179. W196 is located at the turn between β5 and the longest helix, α7. SVD analyses of the intrinsic fluorescence spectra of UCH-L5_N240_ as a function of urea concentration indicate that UCH-L5_N240_ unfolds in a two-state fashion without significant contribution from folding intermediates as in the case for UCH-L1 (Fig. 2). This is further confirmed by far-UV CD spectroscopy that monitors changes in secondary structures. The intrinsic fluorescence and far-UV CD unfolding data were global-fit to a two-state model (Table 1). The free energy of unfolding of UCH-L5_N240_ (9.21 ± 0.39 kcal mol^−1^) is higher than that of UCH-L1 (8.83 ± 0.10 kcal mol^−1^)^33^ and UCH-L3 (7.11 ± 0.20 kcal mol^−1^)^27^, and the transition point of UCH-L5_N240_, [D]_N-D,50%_, is about 0.5 M higher than those of UCH-L1 and -L3 with comparable *m*-values (Table 1).

### Folding kinetics of UCH-L5_N240_

**Figure 3:**
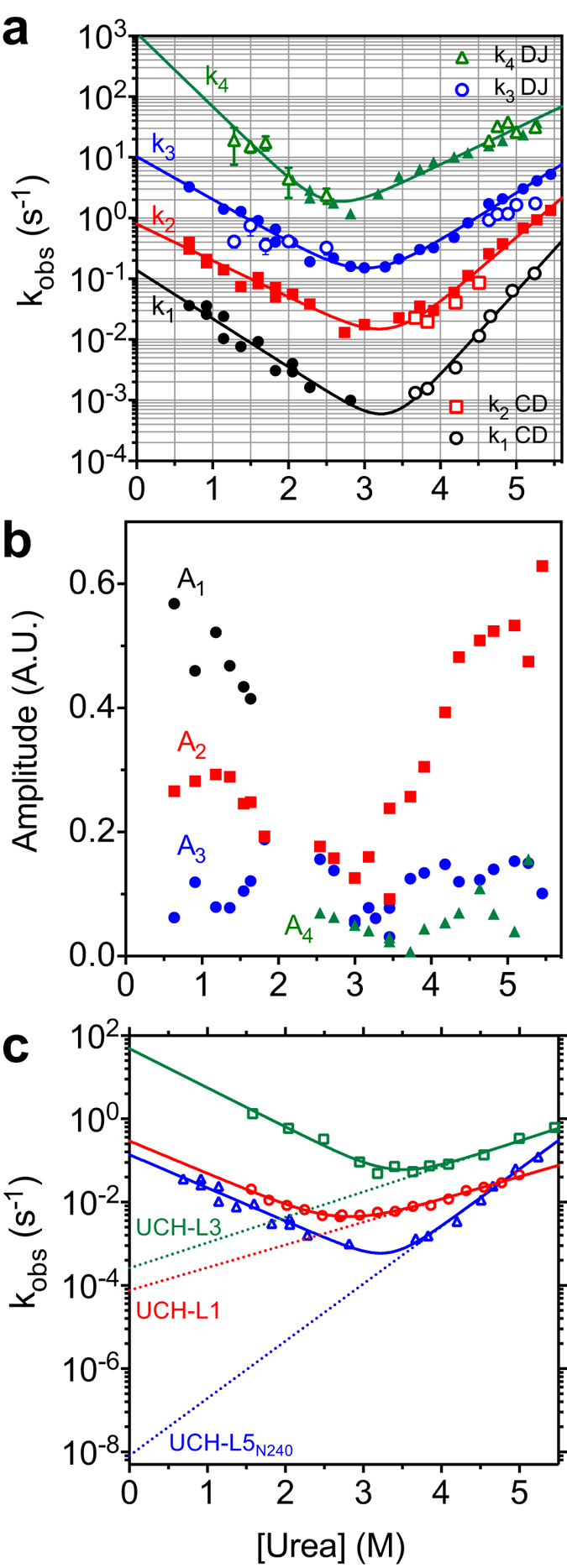
Chevron plot analysis of UCH-L5_N240_. (**A**) The observed reaction rates of the four kinetic phases derived from SJ stopped-flow fluorescence measurements shown in filled symbols. For the two slowest kinetic phases (black and red), some of the unfolding rates were derived from far-UV CD through manual mixing (open symbols). For the two faster kinetics phases, DJ interrupted refolding-based reaction rates are shown in open symbols. The results are fit to a two-state folding model for each kinetic phase and the fitting results are shown in solid lines. (**B**) Amplitudes associated with the four kinetic phases derived from SJ stopped-flow fluorescence measurements. The color-coding are the same as that in panel A. (**C**) Comparison of the slowest kinetic phases of UCH-L1 (red), -L3 (green) and UCH-L5_N240_ (blue). Extrapolations of the unfolding rates as a function urea concentration are shown in dashed line.

We next evaluated the folding kinetics of UCH-L5_N240_ in comparison with those of UCH-L1 and -L3^27^,^28^. Single-jump (SJ) stopped-flow fluorescence measurements of UCH-L5_N240_ as a function of urea concentration yielded four linear refolding arms that we assigned as phases 1, 2, 3, and 4, corresponding to the slowest to the fastest kinetic phases, respectively. The slowest refolding phase 1 has the largest amplitude (*A*_1_), indicating its association with global folding events (Fig. 3). In the case of unfolding kinetics, we only observed three linear unfolding arms that correspond to the kinetic phases 2–4 because the kinetics of phase 1 is too slow to be reliably measured by the stopped-flow mixing device. We therefore used manual mixing followed by far-UV CD measurements (Supplementary Results) to extract two unfolding kinetic phases, with the faster one being consistent with the kinetic phase 2 up to 4.4 M urea; above 4.4 M urea, the kinetic CD traces were fit to a single exponential function due to the limited time resolution to resolve the contribution of the faster kinetic phase. The slower kinetic phase observed in the CD measurements was then associated with unfolding phase 1 (open black circles in Fig. 3a).

By fitting to a two-state model, we obtained four refolding rates and four unfolding rates associated with the four kinetic phases. The fastest and slowest refolding rates were separated by four orders of magnitude, and the fastest and slowest unfolding rates were separated by six orders of magnitude, reflecting a very large dynamic range for the different folding events (Table 2). Amongst the four kinetic phases, the kinetic *m*-value (*m*_*kin*_) and the associated free energy of unfolding of the phase 1 are essentially the same as those derived from equilibrium unfolding (Table 1), while the values for the other three kinetic phases are markedly smaller, suggesting that phase 1 is the dominant term that gives rise to the observed spectral changes in the equilibrium unfolding measurements. In other words, phase 1 reflects the unfolding of the native state (N) into one of the intermediates. Indeed, CD-derived A_1_ associated with unfolding phase 1 is much larger than those of the second slowest phase (Supplementary Results); likewise, SJ fluorescence-derived A_2_ is 4–10 times larger than A_3_ and A_4_ (Fig. 3b).

To compare the folding kinetics of UCH-L1, -L3, and -L5_N240_, we repeated the SJ stopped-flow fluorescence measurements for UCH-L1 and -L3 under the same experimental conditions and focused on the slowest and the most dominant kinetic phases to compare with that of UCH-L5_N240_ (Fig. 3c and Table 3). While the *m*-values associated with the refolding arms of UCHs are similar, the *m*-value associated with the unfolding arm of UCH-L5_N240_ is about twice that of UCH-L1 and -L3, resulting in a much slower unfolding rate in water (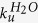 = 8.1 * 10^−9^ s^−1^) that is five and four orders of magnitude slower than those of UCH-L1 and -L3. The folding rate of UCH-L5_N240_ in water (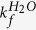 = 0.14 s^−1^) is comparable with that of UCHL1 and is about 100 times slower than that of UCH-L3.

In an effort to establish the relationships between the four kinetic phases in the context of folding pathways, we used double-jump (DJ) interrupted refolding fluorescence analyses to obtain additional refolding rates through global fitting of the kinetic traces obtained with different aging times (*t*_age_) in the presence of different urea concentrations (open blue circles for *k*_3_ and open green triangles for k_4_ in Fig. 3a). Unlike UCH-L1 and -L3, however, amplitude analyses of the kinetic phases 3 and 4 of UCH-L5_N240_ as a function of the aging time (*t*_age_) can be best fit to a linear three-state reaction with the microscopic forward and back reaction rates extracted through global fitting, although the results are noisy and a lag phase in the amplitude build up of the second fastest phase is barely visible. The results therefore suggest the presence of transiently populated intermediate corresponds to the fastest kinetic phase of UCH-L5_N240_ (Supplementary Results). Completion of the kinetic folding pathway of UCH-L5_N240_ for direct comparison with those of UCH-L1 and -L3 will require better instrument stability for kinetic measurements with a timescale that spans over five orders of magnitudes (Fig. 3a).

### NMR HDX reveals abundant local backbone fluctuations in UCH-L5_N240_

Having established the slow global folding kinetics of UCH-L5_N240_, we sought to obtain further structural insights into the origin of stability by solution state NMR spectroscopy. We first obtained near complete assignments of the fingerprint backbone ^15^N-^1^H HSQC spectrum of UCH-L5_N240_, which display highly dispersed cross-peaks corresponding to a well-folded structure, while a group of poorly dispersed cross-peaks with very strong intensities are ascribed to the highly disordered cross-over loop (residues 150–160) and the C-terminal extension beyond the UCH domain (residues 230–240) for which we did not have complete assignments (Supplementary Results). The structural disorder in the cross-over loop, the flanking residues, and the C-terminal region was confirmed by their low order parameters (S^2^) reflecting fast local motions on the ps to ns timescale (Supplementary Results). This timescale however, is more than six orders of magnitudes faster than the fluorescence-based kinetics of UCH-L5_N240_. We therefore resorted to NMR hydrogen-deuterium exchange (HDX) analysis to probe molecular dynamics on a much slower timescale by determining the rate of HDX for a specific amide group and comparing it with the expected value for a random coil to derive the corresponding PF that reflects the stability of the corresponding hydrogen bond (see Material and Methods)^34^.

**Figure 4:**
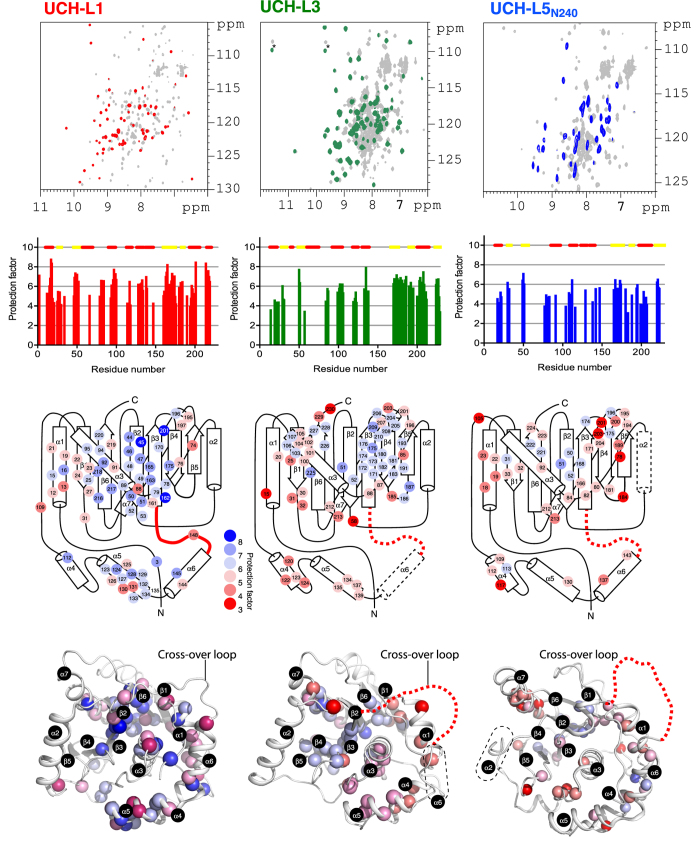
NMR HDX analyses of UCH-L1, UCH-L3 and UCH-L5_N240_. Top panels: Overlaid ^15^N-^1^H HSQC spectra before (grey) and after ca. 30 minutes of HDX (red, green and blue for UCH-L1, -L3 and –L5_N240_, respectively). Aliased cross-peaks in UCH-L3 are indicated by asterisk. Second panels: NMR HDX-derived PFs as a function of residue number. Third panels: Topological mappings of the HDX PFs. The observed PFs are shown in filled circles and coloured from blue to red, corresponding to protection factor values from eight to three as indicted. The numbering of the secondary structures in UCH-L1 (PDB ID: 2ETL) is applied to UCH-L3 and -L5_N240_. Note that α-helix 6 (α6) and α-helix 2 (α2) are absent in the crystal structures of the apo forms of UCH-L3 (PDB ID: 1UCH) and the catalytic domain of UCH-L5 (PDB ID: 3RII), respectively. They are indicated in dashed lines in their respective topological representations. Bottom panels: Structural mapping of the HDX protections factors. The crystal structures of UCH-L3 and -L5_N240_ are superimposed to that of UCH-L1. The backbone amide nitrogen atoms of the residues of which the PFs can be determined are shown in spheres and coloured in the same scheme as that for the topological mappings. The cartoon representations of the crystal structures of UCHs are rendered by PyMol (http://www.pymol.org/).

Contrary to expectations of a slower HDX of UCH-L5_N240_ compared to UCH-L1 and -L3 based on the fluorescence-based equilibrium and kinetic data (Tables 1 and 2), UCH-L5_N240_ exhibits significantly lower PF values, i.e., faster HDX, throughout its backbone amide groups compared to those of UCH-L1 and -L3 (Fig. 4). For instance, α-helix 3 of UCH-L1, which is packed against the central β-sheet as part of the hydrophobic core, is highly protected against HDX, and the C-terminal half of α-helix 3 of UCH-L3 exhibits partial protection against HDX; in contrast, α-helix 3 of UCH-L5_N240_ shows negligible protection against HDX. Likewise, the PFs of the central β-sheet are higher for UCH-L1 than UCH-L3, both of which are higher than the minimal HDX protections of UCH-L5_N240_. In line with the crystallographic findings that indicated highly disordered structures in α-helix 6 for UCH-L3 and α-helix 2 for UCH-L5_N240_, we did not observe appreciable PF for any of the residues within these regions (shown in dashed lines in the top panel of Fig. 4). Consistent with our recent finding of the PUFs of UCH-L1 under native conditions, our current data on UCH-L3 and UCH-L5_N240_ suggest the presence of highly populated PUFs as well: the degree of fluctuations for UCH-L5_N240_ is much higher than that of UCH-L3, and even more so than that of UCH-L1, indicating a high degree of conformational plasticity for UCH-L5_N240_ (Supplementary Results).

### Thermodynamics associated with ubiquitin binding to UCH variants

Earlier studies on the enzyme kinetics of UCH-L1, -L3 and -L5_N240_ have established that both UCHs bind to ubiquitin with sub μM *K*_M_ values^16^,^17^,^18^,^19^,^35^,^36^. While UCH-L1 exhibits higher binding affinity for ubiquitin compared to that of UCH-L3, its hydrolysis turnover rate (*k*_cat_) is about two orders of magnitude slower than that of UCH-L3. We repeated and confirmed the previously reported parameters for UCH-L1, -L3, and -L5_N240_ (Supplementary Results). Although the reported values have a rather broad range of distributions and our *k*_cat_ value is at the higher end, the overall *k*_cat_/*K*_M_ ratios are similar across different reports.

Having established that our recombinant UCHs are enzymatically active, we subsequently carried out isothermal titration calorimetry at four different temperatures (20, 25, 30, and 37 °C) to gain further insights into the ubiquitin binding process (Supplementary Results). For UCH-L1 and -L3, their binding affinities are not sensitive to temperature changes but the enthalpic contributions are clearly more favourable upon increasing temperature. For UCH-L1, the entropic contributions are favourable up to 30 °C and become unfavourable at body temperature, 37 °C. In contrast, for UCH-L3, the entropic contributions become unfavourable at 25 °C. ITC analysis is only applicable to UCH-L1 and -L3 due to the poor ubiquitin binding affinity of UCH-L5; the binding affinity between UCH-L5 and ubiquitin could only be determined by ITC in the presence of Rpn13^6^. Indeed, the addition of 1.2 molar equivalent of ubiquitin slightly increased the thermal stability of UCH-L1 and -L3, raising the melting temperature by 2.2 ± 0.1 °C and 0.9 ± 0.2 °C, respectively, while no significant change in thermal stability was observed for UCH-L5_N240_ (Supplementary Results). The lack of significant thermal stability shift in UCH-L5_N240_ upon the addition of its substrate is consistent with the expectation for weaker binding events^37^. As the CD melting analyses were carried out with only 5 μM protein concentration, the addition of equal molar concentration of ubiquitin may not be sufficient to saturate UCH-L5_N240_. Even after the addition of six-fold higher amount of ubiquitin (30 μM), we did not observe appreciable changes in melting temperature (data not shown). We next carried out NMR titration analyses with 0.1 mM UCH-L5_N240_ and up to 0.15 mM ubiquitin to evaluate the binding processes under conditions that should significantly increase the population of ubiquitin-bound UCH-L5_N240_. The end NMR titration points nonetheless showed severe line-broadening of UCH-L5_N240_ backbone amide and side-chain methyl resonances as opposed to the well-defined backbone and side-chain resonances of ubiquitin-bound UCH-L1^38^, suggesting fast to intermediate exchange processes and therefore unfavourable line-broadening (Supplementary Results).

Despite our inability to obtain a stable binary complex of UCH-L5_N240_ and ubiquitin in solution, we focused on the global analysis of the variable temperature ITC data to extract the heat capacity Δ*C*_p_ in addition to the enthalpy, entropy, and thus free energy associated with ubiquitin binding for the UCH-L1 and -L3 in order to extrapolate the findings to correlate with potential characteristics of ubiquitin binding events for UCH-L5_N240_ (Table 4, Supplementary Results). Under standard conditions, 1 atm and 25 °C, UCH-L1 exhibits the strongest binding affinity towards ubiquitin, with a dissociation constant (*K*_D_) of 0.26 μM, while that of UCH-L3 is significantly weaker (Table 4). While UCH-L1 and -L3 bind to ubiquitin with comparable free energy change, the binding event is entropically favourable at 25 °C in the case of UCH-L1 and entropically unfavourable for UCH-L3, resulting in marked entropy-enthalpy compensation (Supplementary Results). As a result, the changes in heat capacity (Δ*C*_p_) associated with ubiquitin binding is significantly larger for UCH-L3 (−703 cal mol^−1^ K^−1^) compared to that of UCH-L1 (−525 cal mol^−1^ K^−1^). Δ*C*_p_ is associated with the changes in solvent accessible surface area (ΔSASA) upon binding. Indeed, UCH-L3 has a larger ΔSASA value (2691 Å^2^) compared to that of UCH-L1 (2372 Å^2^), according to their respective crystal structures in complex with ubiquitin (PDB ID: 1XD3 and 3KW5, respectively). This is accompanied by a large conformational change and cross-over loop ordering in UCH-L3 manifested in the large entropic penalty associated with ubiquitin binding. It is plausible to speculate that for UCH-L5_N240_, the entropic penalty associated with mono-ubiquitin binding is far greater that that for UCH-L3, hence the much weaker binding that is not accessible to ITC analysis.

## Discussion

In this work, we have characterized in great detail the folding equilibrium and kinetics of the UCH domain of UCH-L5, i.e., UCH-L5_N240_, in comparison with previously reported results on UCH-L1 and -L3. Despite the apparent two-state folding equilibrium behaviour (Fig. 2), four well-resolved kinetic phases induced by urea could be observed by stopped-flow fluorescence measurements, indicating the presence of folding intermediates (Fig. 3). While the four kinetic folding phases have also been observed for UCH-L1 and -L3, and two parallel folding pathways were proposed to connect the native and denatured states, with two distinct folding intermediates being transiently populated along the two parallel pathways^27^,^28^, our interrupted refolding data suggest that the two fastest kinetic phases (*k*_3_ and *k*_4_) are connected sequentially rather than separately with two slower kinetic phases (*k*_1_ and *k*_4_) (Supplementary Results). Subsequent assignments of the connection(s) between the two fastest and two slowest phases were hindered because our stopped-flow instrumentation could not follow the large timescale separating the kinetic phases. The current data nevertheless suggest that the kinetic folding pathways of UCH-L5_N240_ are different from those of UCH-L1 and -L3.

Human UCHs are important DUBs for maintaining cellular proteostasis^2^. Their unique domain architecture – a single domain that binds to and hydrolyses the isopeptide bond of ubiqutinated substrates – with very slow off-rates for releasing hydrolysed ubiquitin products^39^ potentially increase the likelihood of encountering the proteasome machinery that actively unfolds ubiquitinated substrates for degradation. Indeed, it has been suggested by the recent single molecule mechanical unfolding study of UCH-L1 that the mechanical stability of the UCH domain may be beneficial for resisting UPS degradation^40^. Another important finding from the single molecule study is the consistent timescale of refolding triggered from mechanically and chemically unfolded states, implying that the intrinsic unfolding rates in the absence of chemical denaturant can be reliably derived from extrapolation of the linear dependency of chemical denaturant concentration (Fig. 3).

**Figure 5:**
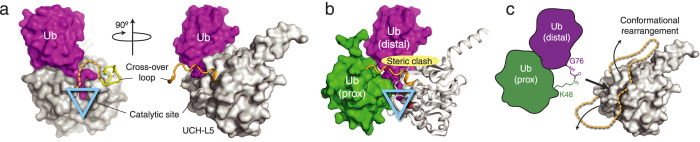
Conformational plasticity of UCH-L5 is required for hydrolysing isopeptide bond of K48-linked di-ubiquitin. (**A**) Orthogonal views of ubiquitin (purple) in complex with UCH-L5 (white) with the cross-over loop being partially disordered (dashed gold line). The location of the catalytic site is indicated by an inverted cyan triangle. (**B**) Model of K48-linked di-ubiquitin in complex with UCH-L5. The proximal ubiquitin is docked to the structure shown in (**A**) through manual rigid-body docking to avoid steric clashes. The cross-over loop is positioned at the interface between the distal and proximal ubiquitin molecules. (**C**) Schematic representation of the conformational rearrangement of the cross-over loop and adjacent structural elements in order to accommodate K48-linked di-ubiquitin.

Our comparative kinetic analysis of UCH-L1, -L3 and -L5_N240_ highlighted the large dynamic range of the intrinsic unfolding rates (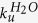) of the most dominant folding events, i.e., unfolding of the native state to one of the intermediate states, amongst the three UCHs. UCH-L5_N240_ has the longest cross-over loop and exhibits the slowest global unfolding rate, four orders of magnitude slower than those of UCH-L1 and -L3 (Fig. 3 and Table 3). The length of the cross-over loop is essential in substrate recognition for UCHs. The longer cross-over loop of UCH-L5 is necessary for binding and hydrolysing the isopeptide bond of K48-linked di-ubiquitin^15^. According to the crystal structure of UCH-L5 in complex with ubiquitin and Rpn13^6^, UCH-L5 binds to mono-ubiquitin in the same pose as other UCHs, but with partially resolved electron density for the cross-over loop (Fig. 5a). However, the cross-over loop and the structural elements around the ubiquitin binding, site such as α-helix 7, need to undergo significant configuration rearrangement in order to accommodate a compact K48-linked di-ubiquitin without steric clashes between the cross-over loop and the substrate (Fig. 5b and c). We deduced an intrinsic structural plasticity from the NMR HDX analysis (Fig. 4), presenting the solution to this issue. Our findings also echo the conformational plasticity implicated in the regulation of the UCH-L5 DUB activity underscored by the recent crystallographic studies of UCH-L5 in complex with different auxiliary factors such as Rpn13 and INO80G for up- and down-regulating the DUB activity of UCH-L5, respectively^6^,^7^.

Generally speaking, longer loop lengths result in higher contact orders and thus slower folding rates^41^. However, the differences in the contact orders between UCH-L1, -L3, and -L5_N240_ are too small to account for the four orders of magnitudes difference in refolding and unfolding rates; the relative contact orders of UCH-L1, -L3, and -L5_N240_ are 0.137, 0.144, and 0.139, respectively (Fig. 3c). The differences in sequence compositions and the folding stabilities of individual secondary structure elements within different UCHs may have more significant contributions to the folding dynamics and kinetics than the contact orders per se. Indeed, despite the abundant internal dynamics within UCH-L5_N240_ in the absence of ubiquitin, it is highly stable thermodynamically and kinetically in terms of chemical stability compared to UCH-L1 and -L3 (Tables 1 and 3). The stabilization effect may be attributed to the higher configurational entropy of UCH-L5_N240_ with respect to that of UCH-L1 and -L3. Indeed, our NMR HDX analysis revealed that the individual secondary structural elements within UCH-L5_N240_ are much less protected from solvent exchange, indicative of abundant local fluctuations (Fig. 4). Rapid HDX in secondary structure elements (mostly peripheral helices) of UCH-L1 has been attributed to highly populated PUFs under native conditions^28^. It is therefore plausible that UCH-L5_N240_ also exhibits abundant PUFs under native conditions in solution, which help stabilize the folding of UCH-L5_N240_.

The extent to which local fluctuations occur within the PUFs of UCH-L5 may be far greater than what was observed in the crystal structure of UCH-L5, which shows structural disorder limited to the cross-over loop in all reported crystal structures and α-helix 2 in the apo form (Fig. 1)^6^,^7^,^11^. Similar loop dynamics was observed in UCH-L5 from *Trichinella spiralis*^42^. Indeed, cross-over loop flexibility was observed in apo UCH-L3, but it was fully resolved in the ubiquitin-form^13^. In contrast, the cross-over loop of UCH-L1 was resolved both in the apo- and ubiquitin-bound forms^9^,^10^. The entropic cost of structural ordering in UCH-L3 is confirmed in our ITC analysis (Table 4 and Supplementary Results). There is a strong correlation between the free energies of unfolding of UCH-L1, -L3 and -L5_N240_ with the lengths of the cross-over loops (Table 3). In addition, the degree of local fluctuations deduced from NMR HDX analysis is proportional to the length of the cross-over loop (Fig. 4).

Using model systems, the effects of loop insertions have been investigated, generally through inserting repeating glycine residues^43^,^44^,^45^. Based on the findings, an empirical function was proposed to probe residual structures in the denatured states with the assumptions that the inserted loop residues marginally destabilize the proteins, that the insertions do not interact with the remaining structure in the denatured, transition, or native states, and that the energetic changes are predominantly entropic^46^. Indeed, such assumptions were met for several other model systems^47^,^48^. Loop elongation however, is not always destabilizing for proteins. A recent study showed that polyglycine loop insertion could in fact significantly increase the entropy of the native state of an acylphosphatase, thereby increasing the native state stability without perturbing the transition and denatured states^49^. In contrast to the artificial manipulation of the loop lengths of model systems, our current results present a biologically relevant example, which has likely evolved to maximize the structural plasticity of UCH-L5 by a longer substrate recognition loop and peripheral helical elements. The structural plasticity therefore contributes to an entropic gain in thermodynamics while the higher flexibility is beneficial for recognizing a broad range of substrates with potentially different ubiquitin linkages without steric clashes with the cross-over loop (Fig. 5). Our NMR titration data (Supplementary Results) strongly suggest that UCH-L5_N240_ remains dynamic in complex with ubiquitin, which may be the underlying reason for its poor *K*_M_ value (Supplementary Results). The entropically stabilized native state of UCH-L5_N240_ significantly reduces the unfolding rate that could be essential for withstanding the potential mechanical unfolding driven by the proteasome when it is associated with the regulatory subunit Rpn13 for DUB activity activation. It is therefore plausible that the functional contribution of the cross-over loops of UCHs that are part of the knotted elements is to balance the need for structural plasticity in substrate regulation and kinetic folding stability that is needed for withstanding proteasome-mediated degradation.

## Methods

### Recombinant protein purification

The pGEX-6P1 vector-based expression construct of UCH-L5_N240_ was a kind gift of Dr. Chittaranjan Das (Purdue University, USA). The resulting recombinant protein construct contains a glutathione *S*-transferase (GST) fused at the N-terminus of UCH-L5_N240_. The expression vector was transformed into a BL21 (DE3) *E. coli* strain and cultured in LB medium containing 0.1 mg/mL ampicillin for antibiotic selection. Recombinant protein over-expression was induced by adding 0.5 mM isopropyl thio-β-d-galactoside (IPTG) when the optical density of bacterial culture at 600 nm (OD_600_) was close to 0.8, followed by overnight incubation with shaking at 16 °C. The cells were harvested and purified by a standard protocol for GST-based affinity column purification, followed by fusion tag removal using PreScission protease (GE Biosciences, USA), as described previously^11^. The resulting recombinant protein was subjected to size-exclusion chromatography using a gel-filtration column (Superdex 75 26/60, GE Biosciences, USA) in 20 mM Tris-HCl (pH 7.6), 100 mM NaCl and 5 mM DTT. The purity of the recombinant protein was higher than 95% judging from SDS-PAGE by visual inspection.

### Intrinsic fluorescence spectroscopy

The intrinsic fluorescence of UCH-L5_N240_ was monitored by exciting the samples at 260 nm and recording the emission spectra between 300 and 450 nm at 25 °C using a temperature-controlled fluorimeter (FP8500, JASCO, Japan). Urea-induced equilibrium unfolding was monitored by generating 41 aliquots of protein solution containing a gradient of urea (0–6 M) concentrations with a linear increment step of 2.5% made by a two-channel liquid dispenser (Hamilton, USA) to minimize manual pipetting errors.

### Far-UV CD spectroscopy

Urea-induced equilibrium unfolding was also monitored by far-UV CD between 200 and 260 nm at 25 °C using a temperature-controlled CD spectrometer (J-815, JASCO, Japan). The spectra were collected with a bandwidth of 1 nm, a data interval of 0.5 nm, and an averaging time of one second. Thermal denaturation of UCH-L5_N240_ was monitored by analysing the CD signal changes at 215 nm between 25 and 80 °C while the changes at 222 nm were monitored for UCH-L1 and -L3. In all cases, 5 μM of protein was used and 1.2 molar equivalent of ubiquitin was added to assess the effect of ubiquitin binding. The resulting isotherms were normalized and fitted to a two-state equilibrium unfolding model to extract the thermodynamics parameters, i.e., melting temperature (T_m_), enthalpy (ΔH), entropy (ΔS) and changes in heat capacity (ΔC_p_) as described previously^50^.

### Thermodynamic parameters of equilibrium unfolding of UCH-L5_N240_ induced by urea

All equilibrium unfolding data were subjected to singular value decomposition (SVD) analysis using an in-house written script for MATLAB (MATLAB and Statistics Toolbox Release 2012b, The MathWorks, USA) as described previously^29^,^30^,^31^. The titration series were used to generate an *m* × *n* matrix, *M*, as inputs for SVD analysis, where *m* corresponds to the number of recorded wavelengths and *n* corresponds to the number of titration points. SVD reconstruction generates an *m* × *m* unitary rotation matrix *U*_r_, an *n* × *n* unitary rotation matrix *V*_r_, and an *m* × *n* diagonal singular value matrix *S* to reconstruct the input matrix *M* as follows


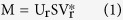


where *U*_r_ corresponds to the basis function of the observed spectral patterns, and *V*_r_ corresponds to the basis coefficient as a function of denaturant concentration. To determine the number of significant components necessary for reconstructing the input matrix, the normalized correlation coefficients of individual singular values were calculated and a threshold of 0.8 was used, leading to the conclusion that UCH-L5_N240_ unfolds in a two-state model.

The fluorescence and CD data were subjected to a two-state equilibrium-unfolding model as described previously^28^,^29^,^30^,^31^. The intrinsic fluorescence and far-UV CD data were fit separately and globally. Additionally, the changes of maximum fluorescence emission wavelength as a function of denaturant concentration were fitted to the same model for comparison.

### Stopped-flow fluorescence spectroscopy

The folding kinetics of UCH-L5_N240_ was monitored using a stopped-flow spectrometer in fluorescence detection mode (SX18 stopped-flow spectrometer, Applied Photophysics, UK) as described previously^28^,^29^,^30^,^31^. Changes in total fluorescence were monitored using an excitation wavelength of 280 nm with a 320 nm cut-off filter. All experiments were carried out at 25 °C. 20 *μ*M UCH-L5_N240_ was buffered in 20 mM Tris-HCl (pH 7.6), 100 mM NaCl with 8 M urea for unfolding or without urea for refolding measurements. For single-jump kinetic measurements, the folding reactions were triggered by rapidly mixing the folding or unfolding buffer with protein solution at an asymmetric mixing ratio of 10:1. After the 11-fold dilution, the final protein concentration was 1.8 *μ*M. For double-jump kinetic measurements, three different modes were employed: interrupted refolding, interrupted unfolding, or sequential refolding kinetic measurement.

For the interrupted refolding measurements, 7.7 M urea-denatured protein solution was first mixed with refolding buffer to initiate refolding over different aging periods, followed by the second mixing step with denaturing buffer containing 8 M urea immediately before we took kinetic fluorescence measurements under denaturing conditions. The first mixing step was achieved by mixing protein solutions with unfolding or refolding buffer with a 1:5 mixing ratio. After a specific aging period, a second mixing step was carried out by mixing the aged protein solution with an equal amount of unfolding or refolding buffer (1:1 mixing ratio) immediately before kinetic fluorescence measurements. The resulting kinetic traces were subjected to global fitting in which the folding rates were shared amongst all the kinetic traces of different aging times, and the amplitudes corresponding to different kinetic phases were shown as a function of aging time, and the rates at which the corresponding amplitudes built up were extracted by single exponential fitting to the observed amplitude as a function of aging time (*t*_age_).

For all kinetic analyses, the observed reaction rates were extracted by fitting the kinetic traces to a single, double, or triple exponential function with an offset using the software package GraphPad Prism (GraphPad Software, USA). The choice of model, *i.e*., number of kinetic phases, was decided using the F-test statistics by Prism.

### Chevron plot analysis

The observed reaction rates in the stopped-flow fluorescence measurements were fit to a simple two-state folding model to extract the associated kinetic and thermodynamic parameters. The observed reaction rates (*k*_obs_), with a linear refolding arm and a linear unfolding arm, were fit to the following equation:


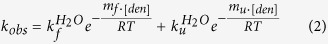


where 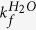 and 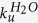 are the folding and unfolding rate in the water, *i.e*., in the absence of denaturant, *m*_f_ and *m*_u_ are the *m*-values associated with folding and unfolding, *R* is the gas constant, and *T* is the sample temperature, which is set to 298 K. These parameters were subsequently used to calculate the free energy of unfolding associated with this reaction as follows:


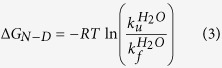


### NMR spectroscopy

Uniformly ^13^C- and ^15^N-labelled UCH-L5_N240_ was obtained by growing the transformed *E. coli* cells in M9 minimal medium containing ^15^N-labelled ammonium chloride (1 g/mL) and ^13^C-labelled D-glucose (2 g/mL) followed by the same purification procedure as described above. For backbone resonance assignments, a suite of triple resonance experiments were collected, including ^15^N-^1^H heteronuclear single quantum correlation (HSQC) spectroscopy, HNCO, HNCA, HNcoCA, HNCACB, and CBCAcoNH at 25 °C. All NMR data were collected using a Bruker AVIII NMR spectrometer, equipped with a cryogen-cooled TCI probe head, operating at 14.1 or 20.0 Tesla, corresponding to a proton Larmor frequency of 600 or 850 MHz, respectively. The data were collected using Topspin 3.0 (Bruker, Germany), processed by NMRPipe^51^, and visualized and analyzed by Sparky (https://www.cgl.ucsf.edu/home/sparky/). Near complete backbone (H, N, C, Cα and Cβ) assignments were achieved through the use of an iterative computer-aided procedure as described previously^52^,^53^. The backbone assignments have been deposited to the BMRB database under the accession number of 12009.

The ubiquitin titration experiment was carried out using 0.1 mM of uniformly ^13^C- and ^15^N-labelled UCH-L5_N240_ with unlabelled ubiquitin (5 mM stock solution concentration) in a 3 mm MATCH tube at 25 °C. and 20.0 Tesla. Two-dimensional ^15^N-^1^H SOFAST-HMQC and ^13^C-^1^H HSQC spectra were collected for individual titration points (0, 0.125, 0.25, 0.375, 0.5, 0.75, 1.0, 1.25, and 1.5 ubiquitin to UCH-L5_N240_ molar ratios) as described previously^54^,^55^.

The ^15^N spin relaxation dynamics of UCH-L5_N240_ was characterized by measuring the longitudinal (R_1_) and transverse (R_2_) relaxation rates and ^15^N-^1^H heteronuclear nuclear Overhauser effects (hetNOEs) of individual backbone amide groups at 37 °C and 14.1 Tesla as described previously^56^. The resulting relaxation rates and hetNOEs were used as inputs to determine the residue-specific order parameters (S^2^) using model-free analysis by TENSOR2^57^. According to TENSOR2, the use of anisotropic diffusion model did not yield statistically significant improvement in the data analysis. We there used a simple isotropic model to analyse NMR relaxation data.

### Hydrogen-deuterium exchange (HDX)

NMR HDX analyses of UCH-L1, -L3, and -L5_N240_ were carried out in 20 mM Tris-HCl (pH 7.6), 100 mM NaCl at 25 °C, as described previously^28^,^33^,^58^. The previously reported backbone assignments of UCH-L3 (BMRB accession number 15121)^59^ were used to confirm the assignments of the ^15^N-^1^H correlations. The amide ^15^N-^1^H cross-peak intensity as a function of HDX time was fit to a single exponential function using the built-in relaxation analysis module of the software package Sparky, and the rate constants were subsequently used to derive the protection factors (PFs) together with the protein sequences using the Excel spreadsheet with pre-defined functions from the Englander laboratory at University of Pennsylvania, USA (http://hx2.med.upenn.edu/download.html).

### Isothermal titration calorimetry (ITC)

A Microcal 200 instrument (GE, USA) was used for the ITC measurements. The protein concentrations of UCHs and human ubiquitin were set to 27.5 and 275 μM, respectively. The latter was used as the titrant to be injected into the UCH solutions. The ITC experiments were carried out at 20, 25, 30, and 37 °C, and the resulting ITC data were individually analysed using NITPIC^60^ and subsequently exported to Sedphat^61^ for global fitting to extract the associated thermodynamics parameters.

### DUB activity analysis

The enzyme kinetics of UCH variants were assessed by UbAMC as a model substrate as described previously^16^,^17^,^18^,^19^. The enzyme concentrations were set to 1 nM, and UbAMC substrate concentrations ranged from 1 to 1000 nM. The fluorescence as a function of time was recorded individually using a temperature-controlled fluorimeter (FP8500, JASCO, Japan). The results were analysed using the built-in Michaelis-Menten equation of the software package GraphPad Prism (GraphPad Software, USA).

## Additional Information

**How to cite this article**: Lee, Y.-T. C. *et al*. Entropic stabilization of a deubiquitinase provides conformational plasticity and slow unfolding kinetics beneficial for functioning on the proteasome. *Sci. Rep.* **7**, 45174; doi: 10.1038/srep45174 (2017).

**Publisher's note:** Springer Nature remains neutral with regard to jurisdictional claims in published maps and institutional affiliations.

## Supplementary Material

Supporting Information

## Figures and Tables

**Table 1:** Thermodynamic parameters of equilibrium unfolding of UCH-L1, -L3 and -L5_N240_ induced by urea.

	UCH-L1^a^	UCH-L3^b^	UCH-L5_N240_		
			Fluorescence	Far-UV CD	Global fit
*m* (kcal mol^−1^ M^−1^)	2.95 ± 0.03	2.28 ± 0.06	2.73 ± 0.08	2.60 ± 0.04	2.61 ± 0.11
ΔG (kcal mol^−1^)	8.83 ± 0.10	7.11 ± 0.20	9.56 ± 0.29	9.05 ± 0.15	9.21 ± 0.39
[D]_50%_ (M)	3.00 ± 0.003	3.12 ± 0.01	3.50 ± 0.01	3.48 ± 0.01	3.53 ± 0.01

^a^The values are taken from ref. 33 that correspond to the native-to-intermediate state transition and were derived from global fitting of fluorescence and far-UV CD data.

^b^The values are taken from ref. 27 derived from fluorescence data fitting to a two-state equilibrium model.

**Table 2:** Kinetic parameters of UCH-L5_N240_ derived from stopped-flow fluorescence measurements.

	*k*_1_	*k*_2_	*k*_3_	*k*_4_
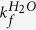 (s^−1^)	0.14 ± 0.03	0.80 ± 0.14	10.3 ± 1.8	1120 ± 740
*m*_f_ (kcal mol^−1^ M^−1^)	−1.09 ± 0.07	−0.82 ± 0.06	−0.96 ± 0.06	−1.65 ± 0.22
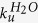 (s^−1^)	(8.1 ± 7.6)e-9	(1.9 ± 1.1)e-6	(3.1 ± 1.2)e-4	(3.1 ± 1.6)e-2
*m*_u_ (kcal mol^−1^ M^−1^)	1.88 ± 0.12	1.41 ± 0.08	1.07 ± 0.05	0.81 ± 0.07
*m*_kin_ (kcal mol^−1^ M^−1^)	2.97 ± 0.14	2.23 ± 0.10	2.03 ± 0.08	1.73 ± 0.14
ΔG (kcal mol^−1^)	9.86 ± 0.57	7.66 ± 0.36	6.16 ± 0.25	6.21 ± 0.50
[D]_50%_ (M)	3.33 ± 0.15	3.44 ± 0.13	3.04 ± 0.10	2.52 ± 0.18

**Table 3:** Comparison of the folding kinetics of UCH-L1, -L3 and -L5_N240_.

	UCH-L1	UCH-L3	UCH-L5_N240_
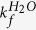 (s^−1^)	0.3 ± 0.1	49 ± 20	0.14 ± 0.03
*m*_f_ (kcal mol^−1^ M^−1^)	−1.06 ± 0.10	−1.28 ± 0.10	−1.09 ± 0.07
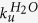 (s^−1^)	(7.6 ± 2.3)e-5	(2.6 ± 0.9)e-4	(8.1 ± 7.6)e-9
*m*_u_ (kcal mol^−1^ M^−1^)	0.74 ± 0.04	0.83 ± 0.03	1.88 ± 0.12
*m*_kin_ (kcal mol^−1^ M^−1^)	1.80 ± 0.11	2.11 ± 0.10	2.97 ± 0.14
ΔG (kcal mol^−1^)	4.90 ± 0.27	7.19 ± 0.32	9.86 ± 0.57
[D]_50%_ (M)	2.72 ± 0.13	3.41 ± 0.13	3.33 ± 0.15

**Table 4:** Thermodynamic parameters of ubiquitin binding to UCH-L1 and –L3.

	UCH-L1	UCH-L3^a^
ΔG (kcal mol^−1^)	−8.99	−8.29
ΔH (kcal mol^−1^)	−5.63	−12.26
-TΔS (kcal mol^−1^)	3.36	−3.98
ΔS (kcal mol^−1^ M^−1^)	11.28	−13.33
ΔC_p_ (kcal mol^−1^ M^−1^)	−0.53	−0.72
K_D_ (μM)	0.26 (0.19–0.35)^b^	0.85 (0.75–0.96)^b^
ΔSASA (Å^2^)		
Non-polar^62^	873	4832
Polar^62^	3531	11130
Non-polar^63^	−6603	−14740
Polar^63^	4426	13658

The thermodynamics parameters were derived by global fitting to the ITC data of UCH variants recorded at 20, 25, 30 and 37 °C using Sedphat^61^. The reported values correspond to the standard condition, i.e., 25 °C.

^a^For UCH-L3, the ITC data at 20 °C were not included in the global fit due to its large errors.

^b^The confidence ranges within one standard deviation are shown in parentheses.
