# Supplementary material for: Entropic stabilization of a deubiquitinase provides conformational plasticity and slow unfolding kinetics beneficial for functioning on the proteasome
Source: Sci Rep. 2017 Mar 24;7:45174. doi: 10.1038/srep45174 (PMC5364529; doi:10.1038/srep45174)
Supplement: Supporting Information [file srep45174-s1.pdf]

Supporting information

Entropic stabilization of a deubiquitinase provides conformational plasticity and slow unfolding kinetics beneficial for functioning on the proteasome

Yun-Tzai Cloud Lee,[1,2] Chia-Yun Chang,[1,2] Szu-Yu Chen,[1] Yun-Ru Pan,[1] Meng-Ru Ho[1] and Shang-Te Danny Hsu[1,2,*]

1. Institute of Biological Chemistry, Academia Sinica, Taipei 11529, Taiwan
2. Institute of Biochemical Sciences, National Taiwan University, 10617, Taiwan

* corresponding author: sthsu@gate.sinica.edu.tw

Supplementary Table S1. Structural comparison of crystal structures of UCH-L5

	3IHR	3A7S	3RII	3TB3	4UEL	4UEM	4UF5
3IHR[1]							
3A7S[2]	0.57						
3RII[3]	0.54	0.36					
3TB3[4]	0.67	0.53	0.58				
4UEL[5]	0.72	0.77	0.74	0.81			
4UEM[5]	0.87	0.69	0.70	0.38	1.04		
4UF5[5]	1.11	0.75	0.72	0.67	1.17	0.62	

Pair-wise positional root-mean-squared deviations (RMSD) in Å of backbone Cα atoms of reported human UCH-L5 catalytic domains. The PDB entries are indicated with their respective references. The results were calculated using PyMol using default setting. 3IHR, 3A7S and 3RII correspond to apo-forms. 3TB3 has six-residue truncation in the cross-over loop. 4UEL corresponds to full-length UCH-L5 in complex with Rpn13 and ubiquitin. 4UEM and 4UF5 corresponds to full-length UCH-L5 in complex with Rpn13 and INO80, respectively.

Supplementary Table S2. Enzyme kinetics parameters of UCH variants using UbAMC as substrate

Protein	k_{cat} (s^{-1})	K_M (mM)	k_{cat}/K_M 10^6 (M^{-1} s^{-1})	Reference
UCH-L1	0.010	0.034	0.31	[6]
	0.020	0.040	0.50	[7]
	0.035	0.047	0.74	[8]
	0.174	0.120	1.45	[9]
	0.033	0.170	0.19	This study
UCH-L3	8.1	0.039	208	[10]
	18.6	0.077	242	[8]
	9.1	0.05	182	[11]
	21.7	0.088	245	This study
UCH-$L5_{N240}$	33.7	21.49	1.57	[8]
	5.5	3.73	1.47	This study

Supplementary Table S3. Thermodynamic parameters of ubiquitin binding to UCH-L1 and -L3 at different temperatures

Protein	Temp (ºC)	n	ΔH (kcal mol^{-1})	ΔS (cal mol^{-1} K^{-1})	K_D (mM)
UCH-L1	20	1.05±0.02	-2.35±0.10	23.5	0.13
	25	1.07±0.01	-4.75±0.09	14.4	0.23
	30	1.15±0.005	-7.05±0.04	6.76	0.28
	37	1.15±0.007	-10.29±0.09	-3.82	0.38
UCH-L3	20	0.71±0.007	-8.00±0.01	2.8	0.27
	25	1.06±0.009	-10.88±0.01	-8.0	0.61
	30	1.05±0.02	-13.63±0.03	-17.4	0.94
	37	1.00±0.02	-16.61±0.04	-26.8	1.44

Supplementary Table S4. Thermodynamic parameters of thermal unfolding of UCHs variants monitored by far-UV CD spectroscopy

Protein	T_m (ºC) Ubiquitin			ΔH_m (kcal mol^{-1}) Ubiquitin			ΔS_m (cal mol^{-1} K^{-1}) Ubiquitin			ΔC_p (kcal mol^{-1} K^{-1}) Ubiquitin		
	-	+	ΔT_m	-	+	$\Delta\Delta H_m$	-	+	$\Delta\Delta S_m$	-	+	$\Delta\Delta C_p$
UCH-L1	57.7±0.1	59.9±0.1	2.2±0.1	128.2±3.7	139.5±4.4	2.3±1.4	2222±65	2328±74	112±58	-1.02±2.40	-1.08±3.07	-0.05±3.90
UCH-L3	62.2±0.1	63.1±0.1	0.9±0.2	81.2±2.4	75.2±2.8	-0.9±1.4	1304±38	1192±45	-106±98	-1.17±0.90	-1.09±0.99	-0.08±1.34
UCH-L5$_{N240}$	58.9±0.4	58.4±0.4	-0.5±0.5	113.5±20.5	121.8±21.5	8.3±29.6	1928±350	2085±369	157±508	4.41±8.70	5.97±7.97	0.16±11.8

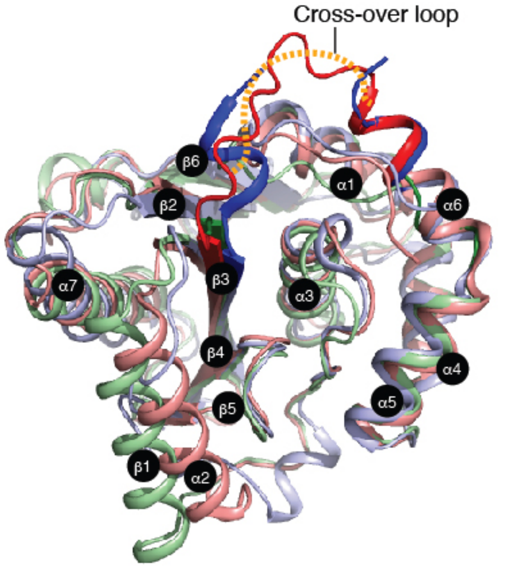


```
UCH-L1 149 EGQCR------VDDKVN 159
UCH-L3 154 EGQTEA---PSIDEKVD 167
UCH-L5 146 QQMFEFDTKTSAKEEDA 162
```

Supplementary Figure S1. Structural alignment of UCH-L1, -L3 and -L5$_{N240}$. The crystal structures of apo UCH-L1 (PDB entry: 2ETL), -L3 (PDB entry: 1UCH) and -L5$_{N240}$ (PDB entry: 3RII) are superimposed and coloured in salmon, pale green and light green, respectively. The cross-over loop and flanking residues of UCH-L1 and -L5$_{N240}$ are coloured in red and blue, respectively, while those of UCH-L3 were not resolved in the crystal structure due to intrinsic disorder. Sequence alignment of the cross-over loops of the three UCHs are shown below.

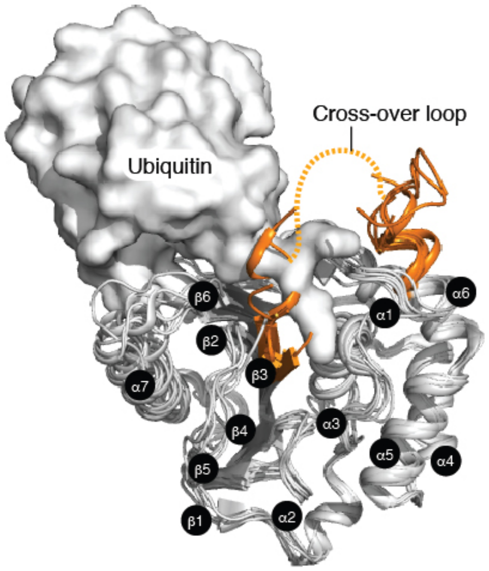


```
     140       150       160
     |         |         |
     HNSFARQQMFEFDTKTSAKEEDAFHF

3IHR HNSFA-----------------AFHF
3A7S HNSFAR----------------AFHF
3RII HNSFARQ----------AKEEDAFHF
3TBT HNSFAR-----XXXXXX-----AFHF
4UEL HNSFARQQMFEFDT------EDAFHF
4UEM HNSFARQQMFE---------EDAFHF
4UF5 HNSFARQQ------------EDAFHF
```

Supplementary Figure S2. Conformational plasticity of UCH-L5. Superposition of seven reported crystal structures of the UCH domain of UCH-L5 shown in white cartoon representations with the cross-over loop (residues 140-165) shown in gold. Dashed line indicates the structural disorder of the cross-over loop leading to unresolved electron density as indicated in dash symbols in the sequence alignment (right panel). 3TBT contains six-residue deletion in the cross-over loop as indicated in crosses. Ubiquitin in complex with UCH-L5 (PDB entry: 4UEL) is shown in surface representation. The secondary structure elements are labelled in the same nomenclature as shown in Figure 1. In addition to the structural variability in the cross-over loop, a-helix 7 that is in contact with ubiquitin also exhibit abundant structural plasticity in the crystal structures of different states of UCH-L5.

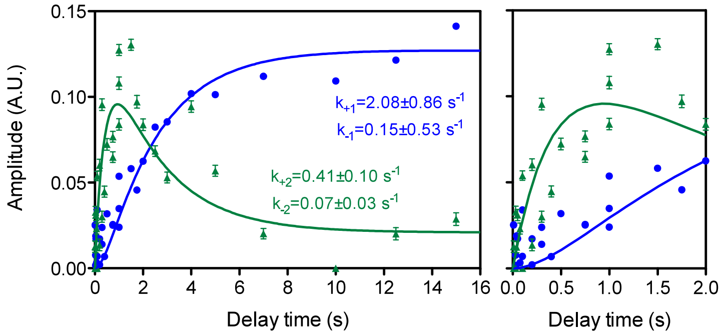

Supplementary Figure S3. Amplitude analysis of interrupted refolding experiments. 7.7 M urea-denatured UCH-L5$_{N240}$ was first mixed with five-fold refolding buffer containing 0.86 M urea and incubated for different delay times to allow refolding, before the second mixing step with equal volume of unfolding buffer containing 8 M urea. The resulting kinetic traces were globally fit to a double-exponential function with shared reaction rates of 26.3 and 1.4 s^{-1}, which correspond to the fastest (green) and second fastest phases (blue), respectively (Figure 3). The associated amplitudes of the two kinetic phases as a function of delay time were fit to a sequential three-state reaction scheme by KinSim. The resulting microscopic forward and reverse reaction rates are indicated next to the fitting results shown in solid lines. Right panel shows the expanded region of the same plot as in the left panel.

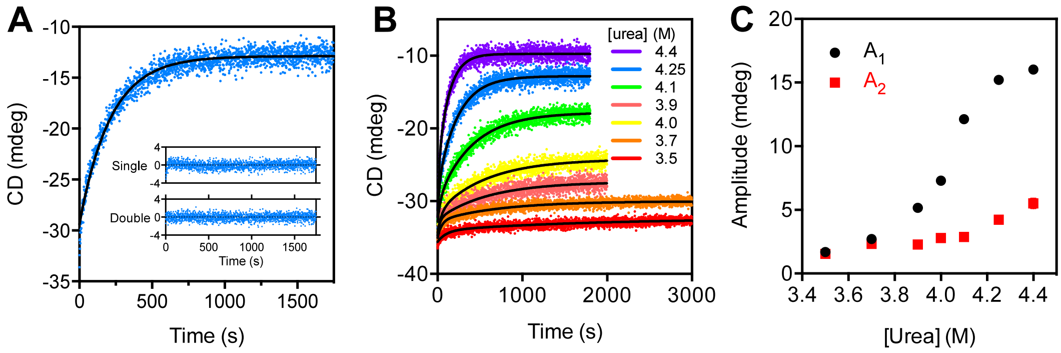

Supplementary Figure S4. Kinetic analysis of urea-induced unfolding of UCH-L5$_{N240}$ by far-UV CD spectroscopy. (A) Kinetic trace recorded in the presence of 4.25 M urea, fit to a double-exponential function as indicated by a solid black line. The residual plots of data fitting to a single- and a double-exponential function are shown in insets. (B) Overview of the kinetic traces recorded after 1:10 manual mixing with native protein with unfolding buffer of different urea concentrations as indicated in the upper right corner. (C) Amplitudes associated with the phases 1 (A_1) and 2 (A_2) observed by stopped-flow fluorescence measurements (Figure 3A).

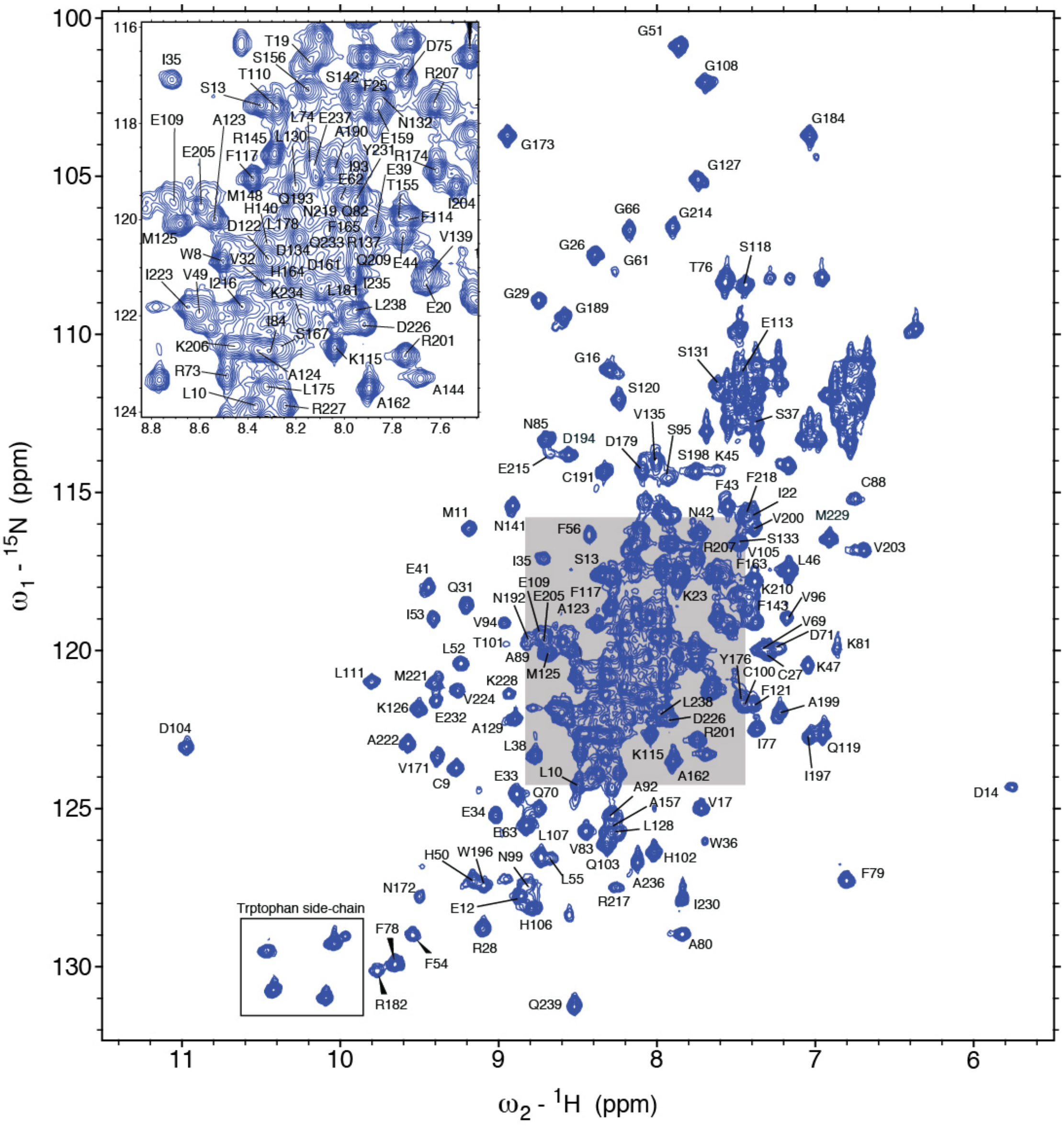

Supplementary Figure S5. ^{15}N-^{1}H HSQC of UCH-L5$_{N240}$ recorded at 25 ºC, 14.1 Tesla. The horizontal and vertical axes correspond to the ^{1}H and ^{15}N chemical shifts, respectively.

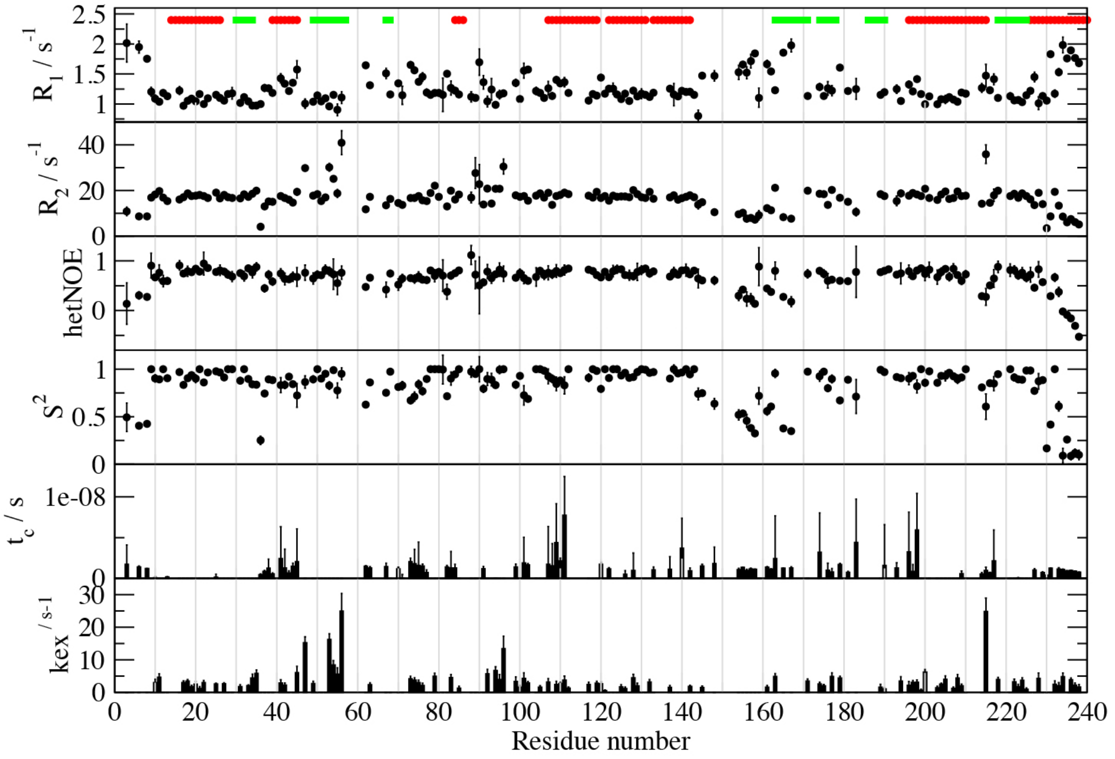

Supplementary Figure S6. ^{15}N spin relaxation analysis of UCH-L5$_{N240}$. An overall rotational correlation time τ_c of 11.83 ± 0.02 ns was used to derive the order parameters of individual residues using an isotropic model by TENSOR2.

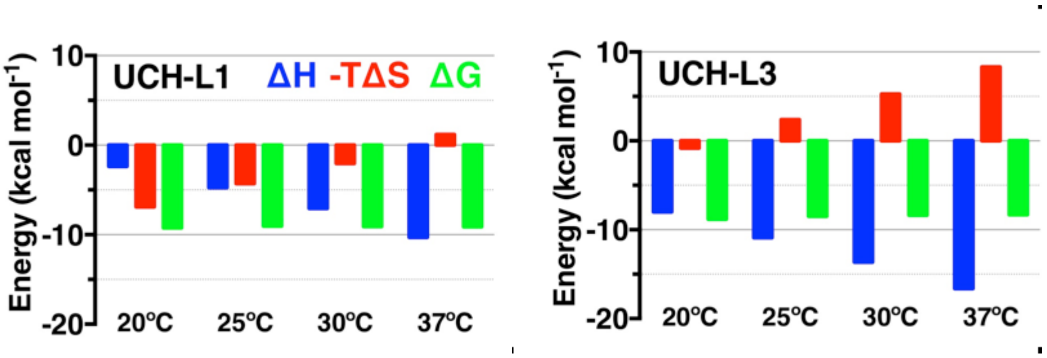

Supplementary Figure 7. Thermodynamic parameters of ubiquitin binding to UCH-L1 and -L3 at different temperatures. Four independent ITC experiments were carried out at 20, 25, 30 and 37 ºC. The associated enthalpy (ΔH), entropy (-TΔS) and free energy ΔG are shown in blue, red and green bars, respectively.

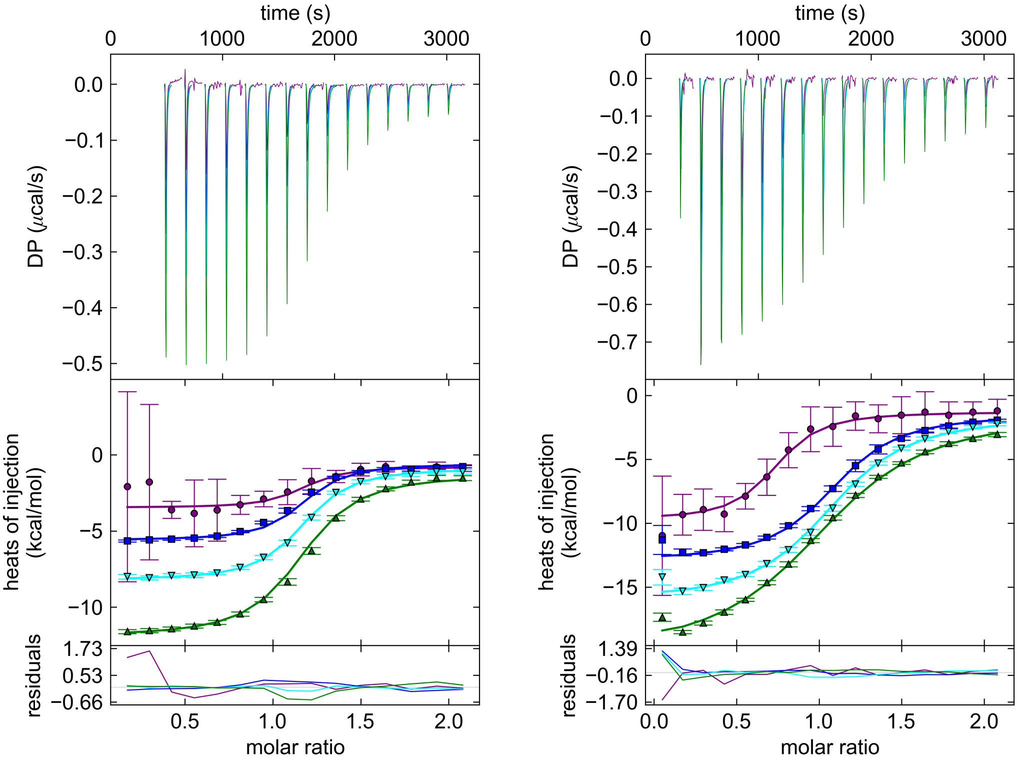

Supplementary Figure S8. Global fitting of ITC data of ubiquitin titration into UCH-L1 (left) and UCH-L3 (right). The titrations were carried out at 20 (purple), 25(blue), 30 (cyan) and 37 (green) ºC. The errors of individual titration points the resulting ITC data were analysed using NITPIC[12] individually and subsequently exported to Sedphat[13] for global fitting using an one-site binding model with an assumption of an 1:1 stoichiometry (n=1) to extract the associated thermodynamics parameters.

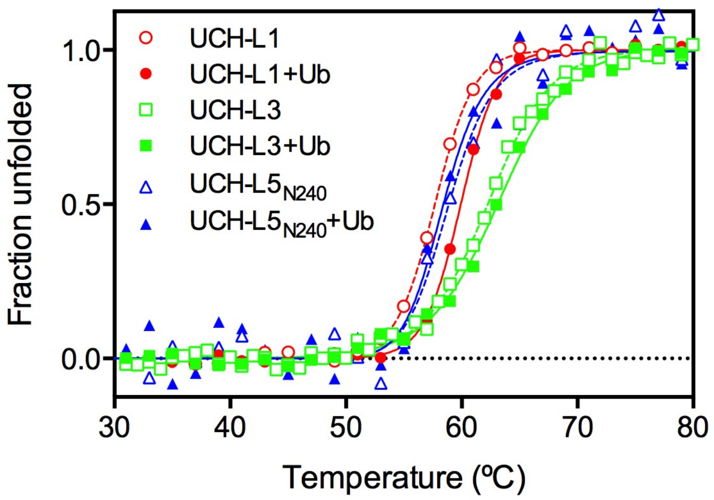

Supplementary Figure S9. Fraction of unfolded UCH-L1, -L3 and -L5$_{N240}$ monitored by far-UV CD spectroscopy. For UCH-L1 and -L3, the CD signals at 222 nm were used; for UCH-L5$_{N240}$, the CD signals at 215 nm were used. To evaluate the effect of ubiquitin binding to the thermal stability of UCH variants, 1.2 molar equivalent of ubiquitin was added for independent measurements.

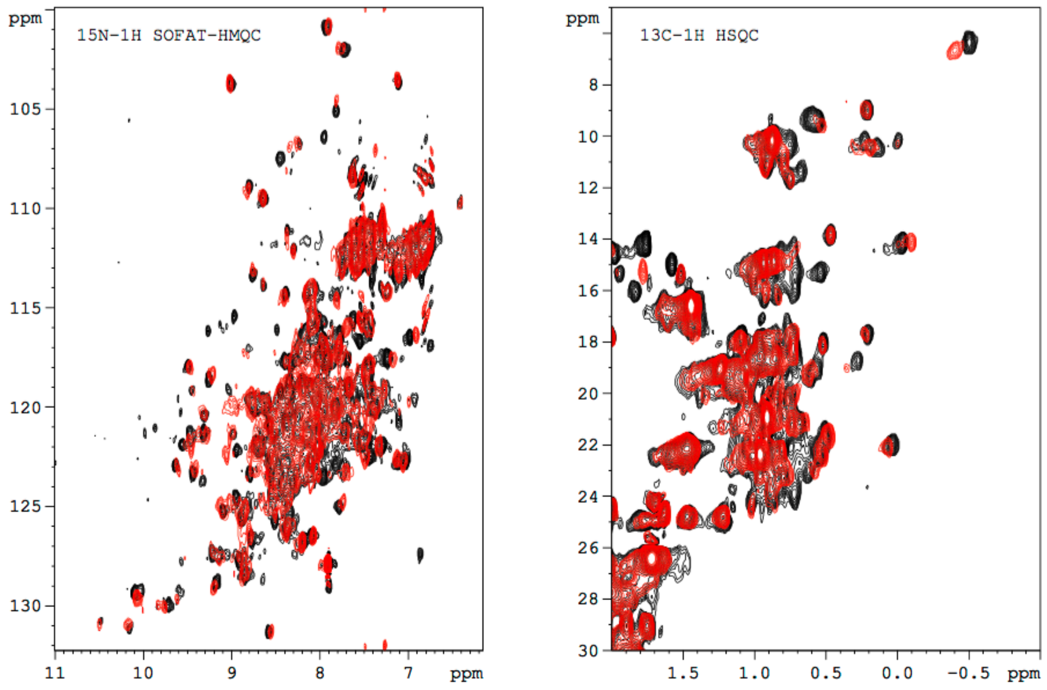

Supplementary Figure S10. Ubiquitin binding to UCH-L5$_{N240}$ monitored by heteronuclear NMR spectroscopy. The binding was monitored at backbone and side-chain level using ^{15}N-^{1}H SOFAST-HMQC (Left) and ^{13}C-^{1}H HSQC (Left) spectroscopy. The spectra of UCH-L5$_{N240}$ without and with 1.5 equivalent of unlabelled ubiquitin are shown in black and red, respectively. Most of the affected correlations showed significant line-broadening with marginal chemical shift changes, indicative of intermediate exchange processes on the timescale between μs and ms.

References

1. Burgie, S.E., Bingman, C.A., Soni, A.B. & Phillips, G.N., Jr. Structural characterization of human Uch37. *Proteins* (2011).
2. Nishio, K. et al. Crystal structure of the de-ubiquitinating enzyme UCH37 (human UCH-L5) catalytic domain. *Biochem. Biophys. Res. Commun.* **390**, 855-60 (2009).
3. Maiti, T.K. et al. Crystal structure of the catalytic domain of UCHL5, a proteasome-associated human deubiquitinating enzyme, reveals an unproductive form of the enzyme. *FEBS J.* **278**, 4917-26 (2011).
4. Zhou, Z.R., Zhang, Y.H., Liu, S., Song, A.X. & Hu, H.Y. Length of the active-site crossover loop defines the substrate specificity of ubiquitin C-terminal hydrolases for ubiquitin chains. *Biochem. J.* **441**, 143-9 (2012).
5. Sahtoe, D.D. et al. Mechanism of UCH-L5 activation and inhibition by DEUBAD domains in RPN13 and INO80G. *Mol. Cell* **57**, 887-900 (2015).
6. Case, A. & Stein, R.L. Mechanistic studies of ubiquitin C-terminal hydrolase L1. *Biochemistry* **45**, 2443-52 (2006).
7. Luchansky, S.J., Lansbury, P.T., Jr. & Stein, R.L. Substrate recognition and catalysis by UCH-L1. *Biochemistry* **45**, 14717-25 (2006).
8. Boudreaux, D.A., Chaney, J., Maiti, T.K. & Das, C. Contribution of active site glutamine to rate enhancement in ubiquitin C-terminal hydrolases. *FEBS J.* **279**, 1106-18 (2012).
9. Nishikawa, K. et al. Alterations of structure and hydrolase activity of parkinsonism-associated human ubiquitin carboxyl-terminal hydrolase L1 variants. *Biochem. Biophys. Res. Commun.* **304**, 176-183 (2003).
10. Dang, L.C., Melandri, F.D. & Stein, R.L. Kinetic and mechanistic studies on the hydrolysis of ubiquitin C-terminal 7-amido-4-methylcoumarin by deubiquitinating enzymes. *Biochemistry* **37**, 1868-79 (1998).
11. Ohayon, S., Spasser, L., Aharoni, A. & Brik, A. Targeting deubiquitinases enabled by chemical synthesis of proteins. *J. Am. Chem. Soc.* **134**, 3281-9 (2012).
12. Keller, S. et al. High-precision isothermal titration calorimetry with automated peak-shape analysis. *Anal. Chem.* **84**, 5066-73 (2012).
13. Zhao, H., Piszczek, G. & Schuck, P. SEDPHAT--a platform for global ITC analysis and global multi-method analysis of molecular interactions. *Methods* **76**, 137-48 (2015).
